# Identifying the earliest-occurring clinically targetable precursors of late-onset Alzheimer's disease

**DOI:** 10.1016/j.ebiom.2024.105238

**Published:** 2024-07-12

**Authors:** Bruce M. Cohen, Kai-Christian Sonntag

**Affiliations:** a Harvard Medical School, Boston, MA, USA; b Program for Neuropsychiatric Research, McLean Hospital, 115 Mill St., Belmont, MA 02478, USA; c Laboratory for Translational Research on Neurodegeneration, Program for Neuropsychiatric Research, McLean Hospital, 115 Mill St., Belmont, MA 02478, USA

**Keywords:** Late-onset Alzheimer's disease, Induced-pluripotent-stem cells, Inherent determinants, Prevention

## Abstract

Most cases of Alzheimer's disease (AD) are late-onset dementias (LOAD). However, research on AD is predominantly of early-onset disease (EOAD). The determinants of EOAD, gene variants of APP and presenilin proteins, are not the basic precursors of LOAD. Rather, multiple other genes and associated cellular processes underlie risk for LOAD. These determinants could be modified in individuals at risk for LOAD well before signs and symptoms appear. Studying brain cells produced from patient-derived induced-pluripotent-stem-cells (iPSC), in culture, will be instrumental in developing such interventions. This paper summarises evidence accrued from iPSC culture models identifying the earliest occurring clinically targetable determinants of LOAD. Results obtained and replicated, thus far, suggest that abnormalities of bioenergetics, lipid metabolism, digestive organelle function and inflammatory activity are primary processes underlying LOAD. The application of cell culture platforms will become increasingly important in research and also on LOAD detection, assessment, and treatment in the years ahead.

## Introduction

Alzheimer's disease (AD), which accounts for 60%–80% of dementias, is typified cognitively by increasing deficits of memory and spatial orientation and structurally by neuronal loss and brain shrinkage.^1^ AD is also associated with toxic beta-amyloid (Aβ) oligomers and hyperphosphorylated-Tau proteins (hpTau), forming senile plaques (SPs) and neurofibrillary tangles (NFTs), respectively, in brain.^2^

Individual cognitive and structural changes and age of onset of AD are variable.^2^ AD before age 65 is uncommon and usually associated with highly penetrant single-gene variants of the amyloid precursor protein (APP) or presenilins (PSEN1 or PSEN2) leading to accelerated accumulation of Aβ. Cases of ‘Early-Onset Alzheimer's disease’ (EOAD) cluster in families and EOAD is sometimes called familial AD. We focus on common forms of AD, occurring at greater prevalence in every decade after age 65, and not associated with APP and PSEN gene variants. Late onset Alzheimer's disease (LOAD) is sometimes called sporadic, but it is associated with genetic predisposition, with an estimated heritability of 60%–80%, and may occur in multiple family members.^3^ LOAD represents about 95% of all AD and over half of all dementias.

In early symptomatic AD, long-axon cholinergic neurons are prominently affected, and for decades, standard treatments were acetylcholinesterase inhibitors, which enhance cholinergic neurotransmission. However, cholinesterase inhibitors have only modest therapeutic effects, along with notable peripheral and off-target side effects.^4^ More recent is the development of antibodies designed to target and remove Aβ. But they, too, have modest therapeutic effects and substantial, even life-threatening, side effects.^4^ New approaches to reduce plaques and tangles, including testing antibodies to hpTau, are being explored. And other causes or concomitants of neurodegeneration, specifically heightened inflammation and mitochondrial deficits, are receiving attention as therapeutic targets.^2^ None of these attempts have yet revealed a treatment with clear therapeutic effects.

Thus far, those receiving treatments have signs and symptoms, and none of the agents used are reparative for damage that has occurred from underlying pathological mechanisms.^2^^,^^4^ Where possible, reducing risk of illness or preventing illness are more desirable interventions than treating existing illness.^4^ Since LOAD appears late in life, there is time to reduce risk if early precursors of disease are detectable and can be modulated.

Inherent factors underlying risk for LOAD include numerous genetic determinants and multiple cellular processes.^5^^,^^6^ These are often studied in the context of early symptoms, but some are abnormal from the start of life and can be explored effectively *in vitro*.^7^ Translational preclinical and clinical studies of promising interventions will follow.^8^

## Genes associated with risk for AD

The most basic inherent determinants of AD are genes associated with illness.^2^^,^^6^ Genetic precursors for EOAD usually include single gene variants in APP and PSEN1/2, and the pathway to neurodegeneration goes largely through misprocessing of APP, accumulation of Aβ oligomers, and formation of SPs. Production of NFTs is partly a downstream effect of toxic Aβ oligomers. Other factors, while they may alter the onset or course of illness, do not ultimately determine much of the risk of EOAD.

For LOAD, as opposed to EOAD, causative roles for Aβ and hpTau are less clear. SPs and NFTs occur in LOAD, but also in normal ageing. 20%–40% of non-demented elderly individuals have neuropathological changes similar to those seen in LOAD.^9^

The most common single-gene variants associated with LOAD are in APOE. About 25% of people carry one copy of *APOE4*, while 2–3% carry two copies. By comparison, 40%–65% of patients with AD carry the APOE4 gene. APOE4 is not fully determinative, however, a single copy of E4 increases risk for AD 3–4 fold and homozygosity increases risk 12-fold.^10^ The E3 variant is most common, and the E2 variant is associated with decreased risk for LOAD. The association of the E4 variant with LOAD suggests that altered lipid metabolism is involved in risk. However, the mechanistic path is not simple (see below).

Other single genes associated with LOAD have been observed,^6^^,^^11^ though no common variant is highly determinative of illness. A recent meta-analysis reports 72 gene loci associated with LOAD.^6^ A few variants, specifically, alleles of TREM2, which codes for a purinergic receptor regulating calcium signalling and motility in microglia, and SORL1, a regulator of endosomal trafficking, determine a substantial portion of risk, estimated at 2–4 fold for TREM2^12^ and 12-fold for SORL1.^13^ However, these variants, while suggesting biochemical pathways to LOAD, are rare. In addition to GWAS, cell culture studies (discussed below) identified genes that contribute to risk for LOAD, including RE1 Silencing Transcription Factor (REST),^14^ protein phosphatase 1 (PP1),^15^ polycomb ring finger 1 (BMI1),^16^ Transgelin 3 (TAGLN3),^17^ and Runt-related transcription factor 2 (RUNX2).^18^

Computational and functional gene network analyses have revealed several key pathways or metabolic processes underlying risk. These include lipid/cholesterol metabolism, immune functions of astrocytes and microglia, mitochondrial function and oxidative stress response, glucose metabolism and insulin response, protein processing, including processing of APP and Tau, and interacting elements of phagocytosis, endocytosis, and autophagy, including mitophagy, the digestion of damaged mitochondria.^6^^,^^19^^,^^20^

## Other approaches to identify risk factors

Studies on post-mortem brain tissue or surgical biopsies, imaging studies, epidemiologic studies, and studies of LOAD-associated physiologic markers in CSF and blood have all reported promising findings.^21^, ^22^, ^23^, ^24^, ^25^ They observe anomalies in many pathways of risk suggested by genetics, including abnormalities of lipid metabolism, membranous structures, and sub-cellular organelles, dysregulation of glucose metabolism, mitochondrial dysfunction, abnormalities in immune and inflammatory processes, and vascular pathology.

This concordance is promising. However, the order of appearance and interactions among different risk-determining factors is not well characterised. Defining the earliest departures from health and the cascade of subsequent events would be of great value for identification of individuals at risk and for developing preventive interventions. An approach using cell lines obtained from patients with LOAD has begun to be pursued for these purposes. Such lines, usually fibroblasts from skin biopsies or cells from blood samples, can be analysed directly or reprogrammed to induced pluripotent stem cell (iPSC) lines, then differentiated, *in vitro*, to brain cells. Studying neural precursor cells (NPC) and their terminal derivatives, neurons and glia, can reveal the inherent anomalies underlying subsequent development of LOAD. Since iPSC replicate an embryonic-like state and lack age-associated epigenetic signatures,^26^ their derivatives lack epigenetic markers accrued during life. Any abnormalities observed in these cell lines reflect premorbid factors associated with LOAD, not the consequences of ageing, diet, or experiences, including trauma or infection, or illnesses and their treatment. Cellular abnormalities are true precursors that interact with one another in the context of subsequent events to determine illness. Protective interventions targeting these precursors may be achievable long before neurodegeneration or even premonitory symptoms occur.

## Studies of iPSCs from patients with LOAD

Numerous studies of iPSC lines derived from patients with AD have been published.^27^, ^28^, ^29^ Of note in these compilations, far fewer papers report results using lines from patients with LOAD than patients with EOAD. And most studies emphasise measures of Aβ, though some detect hpTau. Greater focus on LOAD and factors in addition to APOE seems warranted.

As noted, iPSC-derived neurons and glia are terminally differentiated cells. Although they retain some juvenile characteristics,^30^ they represent cell types similar to those in living brain. (Even NPCs, which are immature cells, occur in mature brain.) They lack most epigenetic markers accumulated by brain cells during life.^31^ For this reason, studying iPSC-derived brain cells does not model ageing. Rather, the lines are suitable for studying inherent factors underlying illnesses, including illnesses that occur with ageing. Organoids, produced in some studies, are embryo like as a whole, but they also contain differentiated neurons and glia. Neurons in organoids do not fully replicate those in living brains, even brains at birth,^32^ but they may be more similar to those in living brain than cells in monocultures.^33^

For comparison, genomic studies performed on DNA isolated from patients are widely accepted as identifying gene variants underlying risk for LOAD. Similarly, studying iPSC derived from patients identifies inherent factors associated with risk for LOAD. Neither gene association studies nor studies of iPSC-derived brain cells model ageing. Respectively, their results suggest inherited and inherent factors associated with risk for LOAD with age.

Studies that included lines from patients with LOAD are categorised and summarised in Table 1. Emphasis is placed on identifying the earliest processes underlying LOAD and observing whether the findings replicate across studies.

**Table 1:** Studies using iPSC lines from patients with LOAD and healthy control subjects.

Abnormal factor	NPCs	Neurons	Astrocytes	Microglia	Organoid/multicell	Total studies per factor	Total cell lines per factor
Lipid metabolism			TCW 2022^34^ [8]	TCW 2022^34^ [8]		1	8
Energy metabolism	Ryu 2021, 2022^7^^,^^8^^,^^35^ [9]	Birnbaum 2018^36^ [5] Chen 2018^37^ [5]	Ryu 2021, 2022^7^^,^^8^^,^^35^ [9]	Konttinen 2019^38^ [3]		4	22
Oxidative stress response		Kondo 2013^39^ [2] Hossini 2015^40^ [1] Ochalek 2017^41^ [4] Zhang 2020^42^ [1] Birnbaum 2018^36^ [5] Balez 2016^43^ [1]	Kondo 2013^39^ [2]	Konttinen 2019^38^ [3] Zhang 2020^42^ [1]	Rouleau 2020^44^ [1]	8	18
Glutamatergic stress response		Duan 2014^45^ [1] Balez 2016^43^ [1] Balez 2024^46^ [3]				3	5
Calcium flux		Duan 2014^45^ [1] Balez 2016^43^ [1] Balez 2024^46^ [3]				3	5
Endo-/Phagocytosis Auto-/Mitophagy		Israel 2012^47^ [2] Hossini 2015^40^ [1] Fang 2019^48^ [1] Mei 2023^49^ [3] Verheijen 2022^50^ [3]		Konttinen^38^ 2019 [3] Xu 2019^38^ [2]	Zhao 2020^51^ [10]	8	25
Inflammatory pathway activity		Chen 2018^37^ [5]	Jones 2017^52^ [1] Arnaud 2022^17^ [4]	Konttinen^38^ 2019 [3] Xu 2019^53^ [2] TCW 2022^34^ [8]		6	23
Total studies	1	15	5	4	2		
Total LOAD cell lines	9	32	24	14	11		

Reports are organised by cell type or multicell culture (columns) and the abnormal factor observed (rows). In the boxes, studies are identified by first author and superscript citation, followed by the number of LOAD iPSC lines used in each study, in brackets. Totals of studies of each cell/culture type and LOAD lines used are provided at the bottom of each column. Total of studies of each factor and total of LOAD lines used in those studies are provided at the end of each row.

Most work has been on iPSC-derived neurons. There are reports on NPCs, astroglia, or microglia. Although oligodendrocytes participate in the development of LOAD,^54^^,^^55^ only one study investigated oligodendrocytes.^18^ A few investigations used spheroids, organoids, or scaffold-based 3-D cultures, with the latter two models containing multiple interacting cell types.^44^^,^^51^ One study produced epithelial cells and astrocytes to model the blood brain barrier and its interaction with brain cells.^56^ All studies in Table 1 include lines from healthy control subjects. Some had comparison lines from patients with EOAD. Those subjects bore variants of APP or PSEN1. Most investigations contained small numbers of independent lines, ranging from 1 to 16 per subject group. One study, designed to define genetic determinants of Aβ production in LOAD, examined 102 lines, but lacking control lines, its results cannot be used to determine differences between LOAD and control cells.^57^ Experiments including control lines used, on average, only 2.5 lines from patients with LOAD, too few to be more than exploratory. The majority of studies reprogrammed fibroblast lines, but some used peripheral blood mononuclear cells (PBMC). Most used protocols that mimic normal development; specifically, iPSC differentiated to NPCs further differentiated to brain cell types. However, several neuronal studies genetically engineered iPSC or NPCs to overexpress neurogenin 2 (NGN-2), causing forced neurogenesis.

### Findings, by culture type, on factors underlying LOAD

Studies on *NPCs* revealed abnormalities in energy production, cell proliferation, and neurogenesis.^7^^,^^14^^,^^35^ One study reported early neuronal differentiation of LOAD NPCs and disrupted nuclear lamina structure, which was partly attributed to diminished nuclear REST, a transcriptional repressor and central regulator of neuronal differentiation.^14^ Another study reported decreased neurogenesis due to induction of a senescence state in NPCs and altered BMP4-SMAD1/5/9-RUNX2 signalling during neuronal cell development in LOAD-derived lines.^18^

Common findings in *neurons* produced from iPSC of patients with LOAD included disturbances in lipid, energy, and RNA metabolism, as well as altered calcium signalling^46^ and accelerated neuronal fate commitment and maturation, with altered synaptogenesis or synaptic vesicle regulation.^14^^,^^43^^,^^58^^,^^59^ Diminished neurogenesis was sometimes but not always observed.^15^^,^^18^ Other findings included deficits in endocytosis, phagocytosis, autophagy or mitophagy,^39^^,^^40^^,^^47^, ^48^, ^49^, ^50^ and increased vulnerability to toxic factors, such as oxidative,^36^^,^^39^, ^40^, ^41^, ^42^^,^^46^ glutamate-induced,^43^^,^^45^^,^^46^ and other stresses.^36^^,^^39^, ^40^, ^41^, ^42^, ^43^^,^^45^, ^46^, ^47^, ^48^, ^49^, ^50^ There was an indication of reduced excitatory cortical or cholinergic neuronal development, while GABAergic neuron development was increased.^60^ Several studies reported concordance between findings in iPSC-derived neuronal lines and post-mortem results in the same individuals. This includes Verheijen et al.^50^ observing abnormalities in metabolism of cholesterol and other lipids in cortical neuron lines, and Balez et al.^46^ observing abnormalities of NOS and calcium signalling in glutamatergic neurons. Lagomarsino^15^ observed overlapping transcriptional profiles and signalling pathways within cells. Chen et al.^37^ reported proteomics analyses of iPSC-derived neurospheres and post-mortem brain tissue from the same subjects with LOAD that revealed evidence of altered immune response, as well as abnormalities in myelin sheath proteins, mitochondrial activity, and antioxidant pathways.

Studies on *astrocytes* revealed findings similar to those reported in neurons or NPCs, including dysregulated lipid (including cholesterol) metabolism,^34^ energy metabolism,^7^^,^^8^^,^^35^ and increased stress responses.^39^ Abnormal morphological characteristics and an accelerated differentiation from NPCs were also observed in astrocytes from patients with LOAD.^52^ Of note, astrocytes participate in immunologic responses in the brain, and LOAD-associated astrocytes exhibited an inflammatory phenotype.^17^^,^^52^

Regarding *microglia*, LOAD-associated cells exhibited increased stress responses when exposed to oxidative stress or lipopolysaccharides.^42^^,^^53^ Cells from patients with LOAD also had increased lipid (cholesterol) metabolism and catabolism, cytokine production, and phagocytosis, as well as reduced cell migration.^34^^,^^38^^,^^42^^,^^53^

Two studies used *3-D cultures* containing mixed cell populations. One reported accelerated neurogenesis and exacerbated apoptosis in cerebral organoids from LOAD lines.^51^ These abnormalities were synergistically increased in APOE4 carriers. There were indications that LOAD organoids exhibited dysregulated signalling pathways related to ER stress, axonal guidance, inflammatory response, RNA metabolism, and lysosomal stress granule formation. A separate study used 3-D silk-based scaffolds, on which iPSC-derived neural and glial cells were co-cultured for two years.^44^ Markers of mitochondrial stress and cell toxicity, and reduced neuronal electrical activity, were observed comparing one LOAD-derived with one control cell line.

### Studies addressing production of Aβ and hpTau

Several investigations assessed secreted or intracellular amounts and proportions of Aβ and hpTau. Most analysed neurons. Across studies presenting data by individual cell line, elevated Aβ (Aβ_40_, Aβ_42_) or Aβ_42/40_ ratios, or elevated tTau, pTau, or pTau/tTau ratios were only found in 25% or 16% of LOAD lines, respectively.^15^^,^^16^^,^^18^^,^^36^^,^^39^^,^^41^^,^^43^^,^^45^^,^^47^^,^^50^^,^^59^^,^^61^ Elevated levels were more often observed with greater duration of culture, suggesting that increases in abnormal peptides are a consequence of more basic preceding mechanisms. In neurons with increased Aβ, some toxic effects could be found, e.g., aberrant endosomes, synapse alterations, cell stress, increased GSK3b activity, and elevated hpTau, though these may also reflect other LOAD-associated mechanisms, not just the presence of toxic Aβ peptides. Four studies looked for evidence of connections between production of Aβ and hpTau; two observed such connections,^15^^,^^47^ two did not.^16^^,^^59^ In the study using 102 lines from patients with LOAD, no association was observed between APOE genotype and production or ratios of Aβ_42_ and Aβ_40_ in iPSC-differentiated neurons.^57^

In addition to studies in neurons, elevated Aβ _40_ was reported on average of 5 LOAD NPC lines.^14^ Increased Aβ_42/40_ and pTau/tTau ratios were observed in long-term neuron-glia co-cultures.^38^ Elevated Aβ_40_, Aβ_42_, tTau, and pTau and their ratios were reported in organoids from 10 LOAD iPSC lines.^51^ Organoids require longer culture times, allowing secondary or delayed features of development to appear. The organoid study also investigated correlation of abnormal peptide production with APOE genotype. No significant associations of Aβ peptides with APOE were observed.^51^ In contrast, APOE4 genotype correlated with higher pTau/tTau ratios. Also, APOE4 related pTau accumulation was independent of Aβ peptide changes. Isogenic conversion of APOE4 bearing LOAD lines to E3 homozygosity (see below) attenuated apoptosis and increased Aβ and pTau metabolites. Taken together, it appears that production of Aβ and Tau only occurs in differentiated cells from a small fraction of LOAD iPSC lines, and these peptides accumulate later in cell culture. In addition, there is little evidence of a strong functional effect of toxic Aβ peptides on Tau phosphorylation. Rather, hpTau levels seem to be associated with APOE4.

### Studies addressing effects of APOE or other LOAD-associated gene variants in gene-edited cell lines

Studies using APOE-defined iPSC lines to identify the earliest factors underlying LOAD are presented in Table 2. Several investigations converted E3–E4 in lines from healthy subjects or converted E4 to E3 haplotype in lines derived from patients with LOAD. Findings demonstrate that APOE4, compared to APOE3 or E2, has pro-inflammatory and immune-activating effects,^17^^,^^34^^,^^63^^,^^64^ dysregulates bioenergetic and lipid (cholesterol and triacylglycerol) metabolism,^34^^,^^62^, ^63^, ^64^ and disrupts endocytosis.^63^, ^64^, ^65^ APOE4 also directly or indirectly perturbs multiple other cell functions. It is associated with decreased neuronal excitability and astrocytic glutamate uptake, but enhancement of synapse activity, Ca^++^ mobilization, endosome formation, astrogliogenesis, neuronal maturation, GABA neuron degeneration, and phagocytosis. Illustrating the interactions among LOAD precursors, APOE4 appears to regulate LOAD-associated genes, including TREM2^12^ and REST,^14^ while APOE is regulated, in turn, by LOAD risk factors, such as SORL1.^66^ In addition, the E4 haplotype alters APP processing and Aβ metabolism along with increasing both Aβ and hpTau, by partly independent pathways, as seen in LOAD cell studies.^51^^,^^58^^,^^59^^,^^62^, ^63^, ^64^

**Table 2:** Studies focusing on APOE genotype-defined iPSC lines.

Abnormal factor	NPCs	Neurons	Astrocytes	Microglia	Organoid/multicell	Total studies per factor	Total cell lines per factor
Lipid metabolism		TCW 2022^34^ [4]	Lee 2021^62^ [1] Lin 2018^63^ [2] TCW 2022^34^ [4] de Leeuw 2022^64^ [3]	TCW 2022^34^ [4]		4	10
Energy metabolism							
Oxidative stress response							
Glutamatergic stress response							
Calcium flux							
Endo-/Phagocytosis Auto-/Mitophagy		Lin 2018^63^ [2]	Lin 2018^63^ [2] Narayan 2020^65^ [2] de Leeuw 2022^64^ [3]	Lin 2018^63^ [2] Moser 2023^24^ [10]		4	17
Inflammatory pathway activity		TCW 2022^34^ [4]	Lin 2018^63^ [2] Arnaud 2022^17^ [1] de Leeuw 2022^64^ [3]	Lin 2018^63^ [2] TCW 2022^34^ [4] Moser 2023^24^ [10]		4	20
Total studies		2	6	3			
Total LOAD cell lines		6	14	16			

Included are studies that use isogenic APOE lines generated from healthy individuals or from patients with LOAD, as well as results from studies using iPSC lines derived from APOE haplotype carriers. In some studies, a combination of such APOE-specified lines was used. Organization and meaning of the items in the table is the same as in Table 1.

In separate studies, investigators compared microglia derived from healthy individuals homozygous for either the E3 or E4 haplotype. APOE4 was associated with abnormal cell morphology, including shorter processes, and increased transition from a resting to a reactive phenotype.^24^

Gene-edited cell lines allow targeted analyses against an isogenic background, thereby isolating the effects of single gene variants. They also offer alternatives when lines from patients with rare mutations are not readily available, as seen in investigations on SORL1^20^^,^^66^ and TREM2.^67^ However, these reprogrammed cells may not carry other gene variants associated with LOAD risk and required for the development of LOAD. While useful for studying single gene effects, they do not model the complement of genes observed in cells from people with LOAD. Also, while results from studying isogenic iPSC lines imply full penetrance of mutations for some biochemical changes *in vitro*, full penetrance does not generally occur *in vivo*. Rather, various individual genetic backgrounds drive much of the LOAD phenotype.^34^^,^^51^

### Lessons from the studies as a whole

**Fig. 1:**
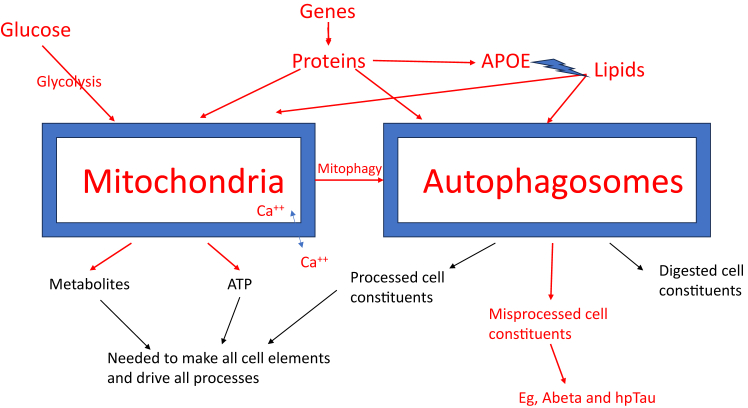
Interrelations of core factors underlying risk and development of LOAD. Schematic of very early appearing abnormalities associated with LOAD. These factors were all reported in studies of iPSC-derived cells from patients with LOAD. Genetic variants underlie these abnormalities, with multiple gene products likely determining each factor. Membranous organelles, mitochondria and autophagosomes, are placed centrally because of their connections with other cellular elements. Arrows indicate production or processes. Red indicates abnormalities observed in the LOAD-associated cells. The blue lightning-bolt arrow indicates that the APOE protein binds and transports lipids. (APOE has other interactions not shown here.) Toxic proteins, such as Aβ and hpTau, are not only produced by abnormal processes associated with LOAD, they also feedback and interact with these same processes, interfering in multiple ways with cell membranes and core functions. Ca^++^ storage and release from mitochondria is shown, but Ca^++^ is also stored and released from other sites. Abnormalities of cell signalling, cycle, growth, and morphology seen in cells derived from patients with LOAD, are higher level anomalies that may largely be a consequence of dysfunctions of the elements shown. External elements are also involved. For example, diet would feed into the model through glucose and lipids, in addition to providing other metabolic precursors. Exercise is known to alter gene expression and resulting cellular metabolism.

Overall, the results suggest that all the factors identified as relevant in past studies of LOAD can be observed and characterised in iPSC-derived cell lines. Specifically, abnormalities in lipid and energy metabolism, stress response, cell growth and differentiation, inflammation, and endocytosis or autophagy/mitophagy have been reported and replicated. In addition, the studies document production of toxic Aβ oligomers and hpTau, notably, in later stages of growth, so they appear to be downstream of the earliest determinants of risk. The appearance of these abnormalities in iPSC-derived lines, even in early neural progenitor cells, suggest they are inherent processes that underly risk and lead to illness in patients, over time and with ageing. An illustration of how these cellular factors may be related is provided in Fig. 1.

## Limitations of the model

As discussed above, cells in culture do not fully replicate the mature growth or interactions of cells in brain. Like genomic studies, findings in cell culture only observe abnormalities associated with LOAD. These variants or processes are not influenced by environment or age, though they may underlie abnormalities that occur during ageing. Identifying such underlying factors is their strength.

Cells from donors, reprogrammed to iPSC lines, and grown out as differentiated lines or composites of lines may accumulate genetic anomalies during culture. Quality control measures limit these occurrences. Studies using new outgrowths of lines or different lines may yield results that are not replicable. LOAD, itself, would appear to include disorders with various underlying causes. In addition, LOAD is a clinical diagnosis, with criteria that may be used differently in different centers. Addressing heterogeneity thoroughly in cell studies requires much larger samples than are currently available.

### Moving towards clinical use

LOAD is a ‘multi-hit’ disorder. Estimating risk and developing interventions requires understanding how inherent, extrinsic, and age-related risk factors are inter-related.^68^^,^^69^ A key feature of LOAD is that accumulation of plaques and tangles are associated with later decades of life and not tightly associated with one another or with illness.^70^ Determinants of pathology may already occur during development and early life, and the factors associated with LOAD are often involved in maintaining brain health throughout life. For example, the brain has very high energy demands, even in a resting state.^71^^,^^72^ Energy production becomes compromised by age-related changes in metabolic activity, including alterations in glucose metabolism and mitochondrial integrity and activity.^73^ Thus, it is not surprising that many of the findings from the iPSC studies directly or indirectly identify abnormalities related to energy metabolism. Similarly, lipid abnormalities feature prominently in LOAD. This includes evidence both of inherent factors underlying altered lipid metabolism, as observed in genomic and cell culture studies, and altered lipid accumulations observed in ageing and post-mortem brain. Not only cholesterols, but also ceramides and other lipids are present at abnormal levels in LOAD.^74^ Membranes are mostly composed of lipids, and membrane composition and integrity are crucial to all brain functions, as all cell processes occur along, across, and within membranes. In this regard, it is notable that alterations of lipids and substantial anomalies of membrane structure and composition have been observed in cells from patients with LOAD. Synthesis and processing of membranous elements is controlled by cell signalling, including calcium flux, and requires energy and the metabolic byproducts from energy production. Importantly, these processes feed back into the composition and function of all cell structures and processes, including membranes. Early determinants of LOAD, such as lipid dysregulation, abnormalities of membranes and membranous elements, as well as energy production and use, could have profound impacts on various other functions found to be abnormal, including autophagy, intracellular signalling, immune responses, and other aspects of cell function and brain health. The consequences of alterations in these risk-associated factors can apparently be tolerated in early life. However, years of ageing provide time for inherent risk factors, as described above, and external or lifestyle risk factors, including diet, exercise, and environment, to interact, resulting in various degrees of neurodeterioration.^68^^,^^75^

SPs and NFTs may be such a common outcome of LOAD-associated factors because accurate processing of proteins, such as APP and Tau, and clearance of their toxic derivatives, depend on the healthy interaction of membranous, energy producing, regulatory, and inflammatory elements. And once Aβ and hpTau form, they cause additional damage to cell membranes and functions. This toxic feedback disposes to neurodegeneration. Further studies on cellular mechanisms may identify control points to target for intervention, inhibiting pathological processes at their source. For example, APP interacts with cholesterols and with lipid rafts containing cholesterols. These cholesterols and lipid rafts, in ER and organelle membranes, may affect APP processing and organelle activity, with broad consequences for cell function and health.^76^ Various genes associated with risk of LOAD, including but not limited to APOE variants, and numerous cell processes alter cholesterol content and distribution in membranes, leading not just to misprocessing of proteins but also to other dysfunctions of membranous organelles, including mitochondria and their energy production by respiration.^77^

Future studies should continue to define genes underlying LOAD-associated anomalies, pathological features observed through imaging and blood work, as well as environmental events that affect risk. But future studies should also prominently include work with iPSC-derived lines. This work can and should be done referencing and in combination with other approaches. Genes of interest can be modified in cell lines, to test the effect of these modifications on cell processes, as has been done in studies of APOE in isogenic lines, as described. In parallel with studies of iPSC-derived lines, which eliminate epigenetic markers, brain cells which retain epigenetic markers can be produced directly from patient samples, by a process called transdifferentiation. Comparing features of iPSC-derived lines and transdifferentiated lines can reveal differences between inherent and age, environment, or treatment related factors. Mouse models can be added to complement genomic and cell culture work. Notably, human cells, derived from iPSC, with or without genetic modifications, can be implanted and studied in various other organs or organisms.^78^

Even alone, cell culture studies have unique strengths for identifying inherent risk factors and discovering control points and targets to normalise processes associated with LOAD. This can be done in predominantly single cell cultures, assemblies of cells from one patient, or in ‘villages’ of cells from multiple donors,^79^ to look for features shared and unique to cell types, individuals, or diagnosis, as a whole. Examples of the early use of cell-culture-based platforms for developing targeted interventions include studying the effects of g-secretase inhibitors, performing compound screens, and testing drugs or other substances *in vitro*.^39^^,^^47^^,^^56^^,^^59^^,^^65^^,^^80^ In addition to preclinical studies, cell platforms can be designed to characterise specific risks for LOAD in individual patients. In time, knowing specific underlying factors in each case could guide the prescription of personalised preventive or ameliorative treatments.^8^

As summarised above, a variety of cell culture systems to study LOAD are already available. All cell types, not just neurons, should be studied, separately and in combinations, as all appear to play a role in determining risk. Multicell systems can mimic cell–cell interactions, such as those between neurons and glia. Organoids are not only multicellular; they can mimic specific areas and structures of the brain. Dual organoids can model inter-regional connections and effects. Other systems under development add features such as vascularity. Too few studies have used these models to study LOAD.

While this review is focused on LOAD, we are aware that there are no clear boundaries among neurodegenerative diseases. This is true clinically, where patients may present with mixed symptoms, for example of LOAD and Parkinson's disease. Also, while the overlap of genetic factors associated with these diseases is modest, most neurodegenerative diseases show some shared abnormalities at the cellular level, including in energy metabolism, autophagy, and inflammation.^81^ Additional information on unique and shared processes underlying these illnesses can be gained by comparing results obtained from iPSC-derived cells of patients with these various diseases.

## Conclusions

Studies must continue on ways to slow the progress of existing AD. Similarly, better ways to support healthy ageing and delay dementia should be sought. But for LOAD, the search for interventions to address the earliest, inherent, determinants of risk for illness should be a high priority. More studies of APOE4 are important, but many cases of LOAD are not associated with APOE4. Efforts to define other factors associated with LOAD and develop other targets for intervention are compelling.

The studies referenced above document likely inherent determinants of LOAD. The concordance between genomic findings and results from reprogrammed cell lines strongly suggest that the factors observed, including abnormal lipid metabolism, energy production, autophagy, and inflammatory processes, are the earliest precursors determining risk for LOAD. Specific interventions to reduce risk can be designed and may lead to prevention of illness on an individual basis, well before neurodegeneration has advanced to a clinical stage.

## Outstanding questions

It will be important to determine how different inherent cellular processes interact with one another to increase or decrease risk for LOAD.

Associations between inherent factors, discovered by genomic and cell culture studies, and later outcomes, including those observed in clinical presentations, by brain imaging, and in post-mortem studies, must be explored. The results will identify how underlying factors are causally linked to outcomes, including ageing and neurodegeneration.

The findings from such studies may reveal where in the path from genetics, through cell processes, to brain structure and function targetable elements lie that could be modulated by drugs, lifestyle, or other interventions to prevent, delay, or slow the development of LOAD.

Collecting data on all the parameters mentioned, inherent, extrinsic, age-related, and clinical, may lead to the identification of subtypes of LOAD, each with different determinants and best addressed by different clinical approaches.

## Contributors

Each author contributed to all aspects of the manuscript, including verifying everything written. Both authors saw all the data, read the entire manuscript, and approved the final version of the manuscript.

## Declaration of interests

The authors have no conflicts of interest related to this manuscript.

## Appendix A Supplementary data

Search strategy and selection criteria

Panel

## References

[1] Alzheimer’s Association (2023). 2023 Alzheimer's disease facts and figures. Alzheimers Dement.

[2] Scheltens P., De Strooper B., Kivipelto M. (2021). Alzheimer's disease. Lancet.

[3] Gatz M., Reynolds C.A., Fratiglioni L. (2006). Role of genes and environments for explaining Alzheimer disease. Arch Gen Psychiatry.

[4] Molchan S., Fugh-Berman A. (2023). Are new Alzheimer drugs better than older drugs?. JAMA Intern Med.

[5] Frisoni G.B. (2023). Complexity is the simple truth about Alzheimer's disease. Lancet Neurol.

[6] Bellenguez C., Kucukali F., Jansen I.E. (2022). New insights into the genetic etiology of Alzheimer's disease and related dementias. Nat Genet.

[7] Ryu W.I., Bormann M.K., Shen M. (2021). Brain cells derived from Alzheimer's disease patients have multiple specific innate abnormalities in energy metabolism. Mol Psychiatry.

[8] Ryu W.I., Cohen B.M., Sonntag K.C. (2021). Hypothesis and theory: characterizing abnormalities of energy metabolism using a cellular platform as a personalized medicine approach for Alzheimer's disease. Front Cell Dev Biol.

[9] Price J.L., McKeel D.W., Buckles V.D. (2009). Neuropathology of nondemented aging: presumptive evidence for preclinical Alzheimer disease. Neurobiol Aging.

[10] Corder E.H., Saunders A.M., Strittmatter W.J. (1993). Gene dose of apolipoprotein E type 4 allele and the risk of Alzheimer's disease in late onset families. Science.

[11] Reitz C., Pericak-Vance M.A., Foroud T., Mayeux R. (2023). A global view of the genetic basis of Alzheimer disease. Nat Rev Neurol.

[12] Gratuze M., Leyns C.E.G., Holtzman D.M. (2018). New insights into the role of TREM2 in Alzheimer's disease. Mol Neurodegener.

[13] Holstege H., van der Lee S.J., Hulsman M. (2017). Characterization of pathogenic SORL1 genetic variants for association with Alzheimer's disease: a clinical interpretation strategy. Eur J Hum Genet.

[14] Meyer K., Feldman H.M., Lu T. (2019). REST and neural gene network dysregulation in iPSC models of Alzheimer's disease. Cell Rep.

[15] Lagomarsino V.N., Pearse R.V., Liu L. (2021). Stem cell-derived neurons reflect features of protein networks, neuropathology, and cognitive outcome of their aged human donors. Neuron.

[16] Flamier A., El Hajjar J., Adjaye J., Fernandes K.J., Abdouh M., Bernier G. (2018). Modeling late-onset sporadic Alzheimer's disease through BMI1 deficiency. Cell Rep.

[17] Arnaud L., Benech P., Greetham L. (2022). APOE4 drives inflammation in human astrocytes via TAGLN3 repression and NF-kB activation. Cell Rep.

[18] Nakatsu D., Kunishige R., Taguchi Y. (2023). BMP4-SMAD1/5/9-RUNX2 pathway activation inhibits neurogenesis and oligodendrogenesis in Alzheimer's patients' iPSCs in senescence-related conditions. Stem Cell Rep.

[19] Andrews S.J., Renton A.E., Fulton-Howard B., Podlesny-Drabiniok A., Marcora E., Goate A.M. (2023). The complex genetic architecture of Alzheimer's disease: novel insights and future directions. eBioMedicine.

[20] Young-Pearse T.L., Lee H., Hsieh Y.C., Chou V., Selkoe D.J. (2023). Moving beyond amyloid and tau to capture the biological heterogeneity of Alzheimer's disease. Trends Neurosci.

[21] Swerdlow R.H. (2018). Mitochondria and mitochondrial cascades in Alzheimer's disease. J Alzheimers Dis.

[22] Kinney J.W., Bemiller S.M., Murtishaw A.S., Leisgang A.M., Salazar A.M., Lamb B.T. (2018). Inflammation as a central mechanism in Alzheimer's disease. Alzheimers Dement (N Y).

[23] Hammond T.C., Lin A.L. (2022). Glucose metabolism is a better marker for predicting clinical Alzheimer's disease than amyloid or tau. J Cell Immunol.

[24] Moser E.D., Manemann S.M., Larson N.B. (2023). Association between fluctuations in blood lipid levels over time with incident Alzheimer disease and Alzheimer disease related dementias. Neurology.

[25] Viejo L., Noori A., Merrill E., Das S., Hyman B.T., Serrano-Pozo A. (2022). Systematic review of human post-mortem immunohistochemical studies and bioinformatics analyses unveil the complexity of astrocyte reaction in Alzheimer's disease. Neuropathol Appl Neurobiol.

[26] Maherali N., Sridharan R., Xie W. (2007). Directly reprogrammed fibroblasts show global epigenetic remodeling and widespread tissue contribution. Cell Stem Cell.

[27] Barak M., Fedorova V., Pospisilova V. (2022). Human iPSC-derived neural models for studying Alzheimer's disease: from neural stem cells to cerebral organoids. Stem Cell Rev Rep.

[28] Hasan M.F., Trushina E. (2022). Advances in recapitulating Alzheimer's disease phenotypes using human induced pluripotent stem cell-based in vitro models. Brain Sci.

[29] Sahlgren Bendtsen K.M., Hall V.J. (2023). The breakthroughs and caveats of using human pluripotent stem cells in modeling Alzheimer's disease. Cells.

[30] Linaro D., Vermaercke B., Iwata R. (2019). Xenotransplanted human cortical neurons reveal species-specific development and functional integration into mouse visual circuits. Neuron.

[31] Scesa G., Adami R., Bottai D. (2021). iPSC preparation and epigenetic memory: does the tissue origin matter?. Cells.

[32] Di Lullo E., Kriegstein A.R. (2017). The use of brain organoids to investigate neural development and disease. Nat Rev Neurosci.

[33] Pasca A.M., Sloan S.A., Clarke L.E. (2015). Functional cortical neurons and astrocytes from human pluripotent stem cells in 3D culture. Nat Methods.

[34] Tcw J., Qian L., Pipalia N.H. (2022). Cholesterol and matrisome pathways dysregulated in astrocytes and microglia. Cell.

[35] Ryu W.I., Shen M., Lee Y. (2022). Nicotinamide riboside and caffeine partially restore diminished NAD availability but not altered energy metabolism in Alzheimer's disease. Aging Cell.

[36] Birnbaum J.H., Wanner D., Gietl A.F. (2018). Oxidative stress and altered mitochondrial protein expression in the absence of amyloid-β and tau pathology in iPSC-derived neurons from sporadic Alzheimer's disease patients. Stem Cell Res.

[37] Chen M., Lee H.K., Moo L., Hanlon E., Stein T., Xia W. (2018). Common proteomic profiles of induced pluripotent stem cell-derived three-dimensional neurons and brain tissue from Alzheimer patients. J Proteomics.

[38] Konttinen H., Cabral-da-Silva M.E.C., Ohtonen S. (2019). PSEN1DeltaE9, APPswe, and APOE4 confer disparate phenotypes in human iPSC-derived microglia. Stem Cell Rep.

[39] Kondo T., Asai M., Tsukita K. (2013). Modeling Alzheimer's disease with iPSCs reveals stress phenotypes associated with intracellular Aβ and differential drug responsiveness. Cell Stem Cell.

[40] Hossini A.M., Megges M., Prigione A. (2015). Induced pluripotent stem cell-derived neuronal cells from a sporadic Alzheimer's disease donor as a model for investigating AD-associated gene regulatory networks. BMC Genomics.

[41] Ochalek A., Mihalik B., Avci H.X. (2017). Neurons derived from sporadic Alzheimer's disease iPSCs reveal elevated TAU hyperphosphorylation, increased amyloid levels, and GSK3B activation. Alzheimer's Res Ther.

[42] Zhang L., Xu M., Ren Q. (2020). Human induced pluripotent stem cell-derived neural cells from Alzheimer's disease patients exhibited different susceptibility to oxidative stress. Stem Cells Dev.

[43] Balez R., Steiner N., Engel M. (2016). Neuroprotective effects of apigenin against inflammation, neuronal excitability and apoptosis in an induced pluripotent stem cell model of Alzheimer's disease. Sci Rep.

[44] Rouleau N., Cantley W.L., Liaudanskaya V. (2020). A long-living bioengineered neural tissue platform to study neurodegeneration. Macromol Biosci.

[45] Duan L., Bhattacharyya B.J., Belmadani A., Pan L., Miller R.J., Kessler J.A. (2014). Stem cell derived basal forebrain cholinergic neurons from Alzheimer's disease patients are more susceptible to cell death. Mol Neurodegener.

[46] Balez R., Stevens C.H., Lenk K. (2024). Increased neuronal nitric oxide synthase in Alzheimer's disease mediates spontaneous calcium signaling and divergent glutamatergic calcium responses. Antioxid Redox Signal.

[47] Israel M.A., Yuan S.H., Bardy C. (2012). Probing sporadic and familial Alzheimer's disease using induced pluripotent stem cells. Nature.

[48] Fang E.F., Hou Y., Palikaras K. (2019). Mitophagy inhibits amyloid-β and tau pathology and reverses cognitive deficits in models of Alzheimer's disease. Nat Neurosci.

[49] Mei T., Li Y., Orduna Dolado A. (2023). Pooled analysis of frontal lobe transcriptomic data identifies key mitophagy gene changes in Alzheimer's disease brain. Front Aging Neurosci.

[50] Verheijen M.C.T., Krauskopf J., Caiment F. (2022). iPSC-derived cortical neurons to study sporadic Alzheimer disease: a transcriptome comparison with post-mortem brain samples. Toxicol Lett.

[51] Zhao J., Fu Y., Yamazaki Y. (2020). APOE4 exacerbates synapse loss and neurodegeneration in Alzheimer's disease patient iPSC-derived cerebral organoids. Nat Commun.

[52] Jones V.C., Atkinson-Dell R., Verkhratsky A., Mohamet L. (2017). Aberrant iPSC-derived human astrocytes in Alzheimer's disease. Cell Death Dis.

[53] Xu M., Zhang L., Liu G., Jiang N., Zhou W., Zhang Y. (2019). Pathological changes in Alzheimer's disease analyzed using induced pluripotent stem cell-derived human microglia-like cells. J Alzheimers Dis.

[54] Maitre M., Jeltsch-David H., Okechukwu N.G., Klein C., Patte-Mensah C., Mensah-Nyagan A.G. (2023). Myelin in Alzheimer's disease: culprit or bystander?. Acta Neuropathol Commun.

[55] Butt A.M., De La Rocha I.C., Rivera A. (2019). Oligodendroglial cells in Alzheimer's disease. Adv Exp Med Biol.

[56] Wasielewska J.M., Chaves J.C.S., Johnston R.L. (2022). A sporadic Alzheimer's blood-brain barrier model for developing ultrasound-mediated delivery of Aducanumab and anti-Tau antibodies. Theranostics.

[57] Kondo T., Hara N., Koyama S. (2022). Dissection of the polygenic architecture of neuronal Aβ production using a large sample of individual iPSC lines derived from Alzheimer's disease patients. Nat Aging.

[58] Wadhwani A.R., Affaneh A., Van Gulden S., Kessler J.A. (2019). Neuronal apolipoprotein E4 increases cell death and phosphorylated tau release in Alzheimer disease. Ann Neurol.

[59] Wang C., Najm R., Xu Q. (2018). Gain of toxic apolipoprotein E4 effects in human iPSC-derived neurons is ameliorated by a small-molecule structure corrector. Nat Med.

[60] Tang Y., Han Y., Yu H., Zhang B., Li G. (2020). Increased GABAergic development in iPSC-derived neurons from patients with sporadic Alzheimer's disease. Neurosci Lett.

[61] Armijo E., Gonzalez C., Shahnawaz M., Flores A., Davis B., Soto C. (2017). Increased susceptibility to Aβ toxicity in neuronal cultures derived from familial Alzheimer's disease (PSEN1-A246E) induced pluripotent stem cells. Neurosci Lett.

[62] Lee S.I., Jeong W., Lim H. (2021). APOE4-carrying human astrocytes oversupply cholesterol to promote neuronal lipid raft expansion and Aβ generation. Stem Cell Rep.

[63] Lin Y.T., Seo J., Gao F. (2018). APOE4 causes widespread molecular and cellular alterations associated with Alzheimer's disease phenotypes in human iPSC-derived brain cell types. Neuron.

[64] de Leeuw S.M., Kirschner A.W.T., Lindner K. (2022). APOE2, E3, and E4 differentially modulate cellular homeostasis, cholesterol metabolism, and inflammatory response in isogenic iPSC-derived astrocytes. Stem Cell Rep.

[65] Narayan P., Sienski G., Bonner J.M. (2020). PICALM rescues endocytic defects caused by the Alzheimer's disease risk factor APOE4. Cell Rep.

[66] Lee H., Aylward A.J., Pearse R.V. (2023). Cell-type-specific regulation of APOE and CLU levels in human neurons by the Alzheimer's disease risk gene SORL1. Cell Rep.

[67] McQuade A., Kang Y.J., Hasselmann J. (2020). Gene expression and functional deficits underlie TREM2-knockout microglia responses in human models of Alzheimer's disease. Nat Commun.

[68] Cohen B.M., Sonntag K.C. (2023). The complex determinants of Alzheimer's-type dementias. Aging (Albany NY).

[69] Butler M., Scott F., Stanton B., Rogers J. (2021). Psychiatrists should investigate their patients less. BJPsych Bull.

[70] Jagust W. (2018). Imaging the evolution and pathophysiology of Alzheimer disease. Nat Rev Neurosci.

[71] Kety S.S. (1950). Blood flow and metabolism of the human brain in health and disease. Trans Stud Coll Physicians Phila.

[72] Ardanaz C.G., Ramirez M.J., Solas M. (2022). Brain metabolic alterations in Alzheimer's disease. Int J Mol Sci.

[73] Cunnane S.C., Trushina E., Morland C. (2020). Brain energy rescue: an emerging therapeutic concept for neurodegenerative disorders of ageing. Nat Rev Drug Discov.

[74] Han X., D M.H., McKeel D.W., Kelley J., Morris J.C. (2002). Substantial sulfatide deficiency and ceramide elevation in very early Alzheimer's disease: potential role in disease pathogenesis. J Neurochem.

[75] Mattson M.P., Arumugam T.V. (2018). Hallmarks of brain aging: adaptive and pathological modification by metabolic states. Cell Metab.

[76] van der Kant R., Langness V.F., Herrera C.M. (2019). Cholesterol metabolism is a druggable axis that Independently regulates Tau and amyloid-β in iPSC-derived Alzheimer's disease neurons. Cell Stem Cell.

[77] Puglielli L., Tanzi R.E., Kovacs D.M. (2003). Alzheimer's disease: the cholesterol connection. Nat Neurosci.

[78] Pomeshchik Y., Velasquez E., Gil J. (2023). Proteomic analysis across patient iPSC-based models and human post-mortem hippocampal tissue reveals early cellular dysfunction and progression of Alzheimer's disease pathogenesis. Acta Neuropathol Commun.

[79] Neavin D.R., Steinmann A.M., Farbehi N. (2023). A village in a dish model system for population-scale hiPSC studies. Nat Commun.

[80] Kondo T., Imamura K., Funayama M. (2017). iPSC-based compound screening and in vitro trials identify a synergistic anti-amyloid β combination for Alzheimer's disease. Cell Rep.

[81] Wainberg M., Andrews S.J., Tripathy S.J. (2023). Shared genetic risk loci between Alzheimer's disease and related dementias, Parkinson's disease, and amyotrophic lateral sclerosis. Alzheimer's Res Ther.

