# Supplementary material for: Identifying the earliest-occurring clinically targetable precursors of late-onset Alzheimer's disease
Source: eBioMedicine. 2024 Jul 12;106:105238. doi: 10.1016/j.ebiom.2024.105238 (PMC11284560; doi:10.1016/j.ebiom.2024.105238)
Supplement: Search strategy and selection criteria [file mmc1.docx]

**Search strategy and selection criteria**

PubMed was searched to find reviews and papers using the search terms:  Alzheimer’s disease combined with other terms relevant to this article, such as human induced pluripotent stem cells or iPSC, cell culture, energy metabolism, glucose and metabolism, autophagy, mitochondria, mitophagy, insulin, inflammation, microbiome, nutrition, fatty acids, genetics, APOE, APP, amyloid beta, Tau, and presenilin.  Recent reviews, notably, several published in the last few years on the use of iPSC lines to study Alzheimer’s disease (AD), were searched for references, as each had slightly different papers cited. For other highly relevant papers, such as reviews of mechanisms underlying AD, the features ‘similar articles’ and ‘cited by’ were also utilized.  Relevant references were often found in the retrieved papers, as well.
