# Supplementary material for: Identifying the earliest-occurring clinically targetable precursors of late-onset Alzheimer's disease
Source: eBioMedicine. 2024 Jul 12;106:105238. doi: 10.1016/j.ebiom.2024.105238 (PMC11284560; doi:10.1016/j.ebiom.2024.105238)
Supplement: Panel [file mmc2.docx]

**Panel: Purpose and content of this Personal View in context**

**The background:** Although most cases of dementia are late-onset Alzheimer’s disease (LOAD), existing research on AD has primarily been focused on early-onset disease (EOAD). The brain of patients with EOAD has numerous senile plaques (SP) and neurofibrillary tangles (NFT), associated with the production of toxic beta-amyloid peptide (Aβ) and hyperphosphorylated-Tau protein (hpTau). These deposits are not consistently found in the brains of patients with LOAD. EOAD is largely determined by inherited variants of genes for the amyloid precursor protein (APP) or presenilins. These highly penetrant gene variants do not underlie LOAD. Instead, gene variants affecting multiple metabolic pathways, including haplotypes of the Apolipoprotein E gene, coding for a lipid transporting protein, are associated with LOAD. It remains unclear how these inherited variants alter cell functions and lead to dementia, late in life. An especially useful technology to find and characterize these inherent abnormalities employs cells obtained from individuals with LOAD. One powerful approach reprograms blood or skin samples, first to induced pluripotent stem cells (iPSC), and then to brain cells. By comparing characteristics of cell lines from patients with LOAD to lines from healthy individuals, investigators can identify the earliest appearing cell processes or factors underlying LOAD. If interventions can be developed to modify these abnormalities, preventive treatments could be developed.

**The key evidence observed:** Investigators have differentiated iPSC lines from patients with LOAD both to individual brain cell types and to assemblies of brain cells. Individual cell types studied have included neurons, astrocytes, oligodendrocytes, and microglia. Multicell associations have included mixed brain cell types, 3-dimensional brain assembloids, and organoids, which model regions of the brain. In most articles, the number of cell lines studied was small. Nevertheless, the studies have identified and replicated several key LOAD associated abnormalities, including anomalies in the production of energy by glycolysis and oxidative phosphorylation, lipid metabolism, digestion of cell contents by phagocytosis, autophagy, or mitophagy, as well as evidence of molecular and cell level alterations in inflammatory activity. Some of these abnormalities correlated with specific APOE or other LOAD associated risk gene variants, consistent with a role for these inherited variants in determining LOAD pathology. Of note, the production of Aβ or hpTau, the elements of SP and NFT observed in the brain in EOAD, were inconsistently seen in cell culture studies of LOAD. In addition, when overproduction of Aβ or hpTau was seen, that finding predominantly occurred in cultures grown for longer periods of time. As a whole, the results obtained from iPSC studies suggest that abnormalities of bioenergetics, lipid metabolism, digestive organelle dysfunction and inflammatory activity are early occurring primary risk processes underlying LOAD. Excessive Aβ or hpTau accumulations appear to be later developing abnormalities. They may be downstream consequences of earlier factors underlying risk for LOAD.

**Implications for further investigation and clinical understanding:** Unlike EOAD, which is usually determined by a single gene variant leading to accelerated accumulation of Aβ and hpTau, LOAD is a ‘multi-hit’ disorder. It is the outcome of the interaction of a variety of factors. Some are determined genetically. Some, of course, occur or are magnified through the aging process. Among key inherent mechanisms and pathways are altered energy and lipid metabolism, along with abnormal inflammatory activity. Attention to diet and exercise, as clinically advised, may modulate these risks. By comparison, intervening with anti-amyloid or anti-tau antibody treatments is after-the-fact. Damage to the brain has already occurred once signs of plaques and tangles or symptoms of cognitive dysfunction appear. Benefits from removing some Ab and hpTau would be expected to be modest, at best, just as observed. Instead, further study characterizing early abnormalities and their control points may lead to targeted interventions that could directly prevent or reduce the damage mediated through LOAD-specific metabolic and cellular risk factors. Because risk factors vary from person to person, best interventions will also vary among those at risk. From a diagnostic standpoint, cell culture platforms, using cell lines from individual patients, may serve as useful tools for identifying individuals at risk and for recommending personalized treatments. Abnormalities documented by such platforms would complement information from genotyping and lifestyle evaluation. Applying these tools to each person at risk could guide interventions most likely to benefit that particular individual. As illustrated by the development and application of premorbid and diagnostic testing to choose targeted interventions and treatments in oncology, such an approach is practical. Its use for assessment and treatment in LOAD will become possible once information on underlying mechanisms of LOAD becomes definitive.
